# Printing Optimization of 3D Structure with Lard-like Texture Using a Beeswax-Based Oleogels

**DOI:** 10.4014/jmb.2209.09052

**Published:** 2022-10-24

**Authors:** Hyeona Kang, Yourim Oh, Nam Keun Lee, Jin-Kyu Rhee

**Affiliations:** Department of Food Science and Biotechnology, Ewha Womans University, Seoul 03760, Republic of Korea

**Keywords:** 3D food printing, beeswax oleogels, alternative fat, texture

## Abstract

In this study, we investigated the optimal conditions for 3D structure printing of alternative fats that have the textural properties of lard using beeswax (BW)-based oleogel by a statistical analysis. Products printed with over 15% BW oleogel at 50% and 75% infill level (IL) showed high printing accuracy with the lowest dimensional printing deviation for the designed model. The hardness, cohesion, and adhesion of printed samples were influenced by BW concentration and infill level. For multi-response optimization, fixed target values (hardness, adhesiveness, and cohesiveness) were applied with lard printed at 75% IL. The preparation parameters obtained as a result of multiple reaction prediction were 58.9% IL and 16.0% BW, and printing with this oleogel achieved fixed target values similar to those of lard. In conclusion, our study shows that 3D printing based on the BW oleogel system produces complex internal structures that allow adjustment of the textural properties of the printed samples, and BW oleogels could potentially serve as an excellent replacement for fat.

## Introduction

Animal meat is a healthy, balanced food group that provides protein, minerals and vitamins. However, meat production and consumption are constrained by issues such as water, land scarcitiy, animal welfare, and climate change [1]. According to a United Nation (U.N.) estimate, the global population will reach 9.7 billion by 2050 [2]. This sharp population and economic growth makes it impossible to decrease the demand for meat products [1]. To alleviate such problems, interest in meat substitues has increased. Since the texture and flavor of meat are greatly influenced by marbling (intramuscular fat) [3], fat is the most important factor to be considered when manufacturing alternative meat. 3D food printing (3DFP), a new technology in the field of food science, is an innovative manufacturing method that allow customized food designs, personalized nutrition, simplified supply chains, and expansion of available food ingredients, such as microalgae and edible insects [4, 5]. Moreover, 3D printing may be an effective technique for producing alternative meats with marbling using fat materials as opposed to existing alternative meat processing methods.

Oleogelation triggered by gelators induces a three-dimensional viscoelastic network in which liquid oil becomes trapped. It is a new and effective fat replacement technique that renders the liquid phase of fat semi-solid structured [6-8]. This process offers both the nutritional benefits of liquid oils [9] and the positive technical and sensory properties of solids [10-13]. Therefore, oleogels have excellent potential as printable materials in 3DFP due to their physicochemical properties that can be customized as desired [14]. Oleogels form thermo-reversible, 3D gel networks at the nano and micro scale and require organic oleogelators to induce a variety of gelation mechanisms that provide specific macroscopic features, such as rheology and texture [7, 12, 15]. The mechanical and physical properties of oleogels are influenced by type, molecular weight, and concentration of oleogelator, the type and polarity of oil, the presence of other additives such as surfactants, and crystallization temperature [16-18].

Beeswax (BW) is a low-molecular-weight oleogelator that forms a network of crystalline oleogelation particles and has only recently been used for oil structuring [7, 19]. Currently, BW has a variety of applications in the food industry, including as a texturizer for chewing gum base, carrier and stabilizer for food additives, and as a clouding, releasing, and glazing agent (for food flavors and colors) [17]. Recognized as food additive E 901, BW is also a globally approved food additive certified by the Food and Drug Administration (FDA, 21 CFR 184.1973) as GRAS (generally recognized as safe) [20, 21]. In a study on oleogels using BW as an oleogelator in meat products, Moghtadaei [19] reported sesame oil based-oleogels as a fat substitute in beef burger. Franco [22] studied linseed oil based-oleogels as a fat counterpart of pork backfat in frankfurter sausages.

Infill structures such as infill patterns and infill levels (IL) are important factors in 3D printing processing. Changes in the infill structure can affect the sensory and texture properties of printed foods by contributing to the stability and strength of the structure by changing the mass and void fraction deposited in the printed inner structure [23, 24]. In this context, complex, digital food design that cannot be achieved with traditional food processing methods can become a reality [5]. However, research on the effect of infill structure on the texture of printed foods is still limited, and current research is limited to studies on 3D printed cheese [25] and mashed potatoes [5].

Therefore, in this study we sought to establish the optimal conditions for 3D food printing of alternative fats that have the textural properties of lard using alternative fats composed of BW and high oleic sunflower oil (HOSO). To this end, 3D printed objects were prepared according to the BW concentration and printing filling level of alternative fat, and the textural properties (hardness, cohesiveness, and adhesiveness) were compared with the printed lard structure.

## Materials and Methods

### Materials

HOSO and lard (Cypro lard oil, Daekyoung O&T, Korea) were purchased from a local grocery store. BW (food-grade, acacia type), a yellow solid cube with a mild odor, was provided by Honest Honey (Korea).

### Production of Oleogels

For the preparation of oleogels, 30 g of HOSO was weighed in a 100 ml Pyrex beaker, and an appropriate amount of BW was added according to the treatment. The mixture was heated to 90 ± 5°C for 30 min under magnetic stirring at 300 rpm. After complete dissolution of the oleogelator, the molten oleogel was poured into a polypropylene syringe (2.2 cm inner diameter). Samples were used after being stored at 23 ± 2°C for at least 24 h [26].

### Experimental Design

A central composite face-centered (CCFC) design was used in the response surface methodology (RSM) method for the analysis of two independent variable effects (BW concentration and IL), interactions, and quadratic terms on the main properties of 3D printed objects [27]. A total of 13 experiments were performed in this work, including 5 replicates at the central point (Table 1). The total number experiments were calculated using the following equation [28]:

$${\text{N = 2}}^{n}+2n+n_{c,}$$ (1)

where *N* is the total number of experiments required; *n* is the number of factors; and *n_c_* is center runs (five replicates).

Previous studies have suggested adding at least 4 center runs for a central composite design, to achieve adequate pure error freedom and reasonably sensitive lack-of-fit testing [29, 30].

The sequence of experiments was completely randomized to minimize unexpected errors in responses due to external factors. BW concentration (11, 15, and 19%) and IL (50, 75, and 100%) were determined from preliminary experimental results (data not shown). Each level was selected by conducting several test experiments taking into account the maximum operating level of the printing equipment.

The dependence of each experimental response on the independent variable was modeled by applying the following second-order polynomial Eq. (2) [28, 30]:

$$Y=\beta_{0}+\sum_{j=1}^{k}\beta_{j}X_{j}+\sum_{j=1}^{k}\beta_{jj}X_{j}^{2}+\sum_{i}\sum_{<j=2}^{k}\beta_{ij}X_{i}X_{j}+e_{i}$$ (2)

where Y is the response; X_i_ and X_j_ are variables (i and j range from 1 to k); *β*_0_ is the constant term; *β*_j_, *β*_jj_, and *β*_ij_ are the interaction coefficients of the linear, quadratic, and second-order terms, respectively; *k* is the number of independent parameters (*k* = 2 in this study); and ei is the error [31, 32].

### Modeling of 3D Design

**Fig. 1:**
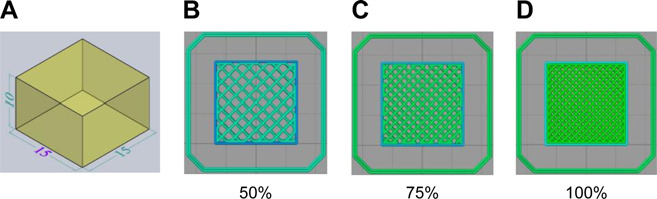
CAD design and a sliced model. A, perspective view of CAD design; B–D, top views of the sliced model with three different printing infill levels.

The model for the 3D structure printing of fats was designed with computer-aided design (CAD) software (SOLIDWORKS 2016) and exported as .stl files. The 3D model was designed as a rectangular prism with the following dimensions: length = 15 mm, width = 15 mm, and height = 10 mm [33]. Next, the generated .stl file was imported into the computer slicing software Simplify 3D (ver. 4.1.1). The internal fill pattern was a grid, and the internal fill density was designed with three different percentages (50, 75, and 100%) (Fig. 1). Based on preliminary experiments, the minimum percentage of the infill level was set to 50%.

### 3D Printing Parameters

The printing process was performed using a model 3D printer (Shinnove, Hangzhou Shiyin Technology, China) with a metal syringe connected to a temperature-controlled heating system with the temperature between 23°C and 100°C, and an interchangeable metal nozzle [33]. The operating parameters of the 3D printer are shown in Table 2. All printing tests were performed using printing parameters of the same value with only temperature parameters being modified to ensure the adequate printability of the sample [14]. All samples were printed at room temperature (23°C).

Printing accuracy was calculated as the difference between the CAD design and experimental dimensions using vernier caliper (Digital Caliper, RUITOOL, China). The designed model was a square, with the same length and width (15 mm × 15 mm). The length and width were measured at three different positions, and the height (10 mm) was measured at five positions at the center and the edge. The dimensional printing deviation in each direction was determined as follows:

$$\begin{array}{l} \text{Dimensional deviation in height (\%) = }\frac{\text{A-B}}{\text{B}}\text{×100;}\\ \text{Dimensional deviation in length and width (\%) = }\frac{\text{C-D}}{\text{D}}\text{×100;} \end{array}$$

where A is the height of the printed sample, B is the height of the designed model, C is the length and width of the printed sample, and D is the length and width of the designed model. Dimensions and weight values of printed samples were recorded after storage at -4°C for 24 h.

### Texture Properties of 3D Printed Products

Sample texture was determined by a texture profile analysis (TPA) of double-cycle compression test using a texture analyzer (TA.XT2i Plus, Stable Microsystems, UK) equipped with a 50 N load cell [34]. Samples in solid form and stored for 1 h at room temperature were placed on a test platform [35]. Samples were compressed every cycle to deform 60% of their original height using a 25 mm square probe at a pre-test and test speed of 1 mm/s. The hold time between compressions was set to 5 s. Next, the probe was returned to its initial position at a pos*t*-test speed of 5 mm/s. Hardness (HA), cohesiveness (CO), and adhesiveness (AD) were recorded on the test curve [33]. HA was determined as the maximum force measured during the first compression cycle in the force-time curve [34]. CO is defined as the ratio of the positive force domain (A3/A1) in the first and second compression cycles [36]. AD is defined as the area of negative force required to detach the compression plunger from the sample in the first compression cycle [37]. Each test was repeated at least five times for each type of sample.

### Statistical Analysis

All results are presented as mean ± SD values. The analysis was performed in triplicate (n=3). One-way ANOVA with Duncan’s test was carried out by SPSS (SPSS, USA). The graph was expressed using Sigma Plot software (version 12.5, USA). In addition, the experimental results of CCFC (central composite face-centered design) were analyzed by Minitab®20 software (Minitab Inc., USA). Response surface analysis modeling analyzed quadratic mathematical models including linear, squared, and interaction terms, and statistical analysis of quadratic models used analysis of variance (ANOVA). The experimental data were evaluated by various descriptive statistical analyses such as *R*^2^ (goodness of fit of the regression model), *F*-value (statistical significance of the overall model), *p*-value, mean sum of squares, degrees of freedom (DF), and sum of squares. When the *p*-value was <0.05, it was considered statistically significant, and the lack-of-fit test was used to evaluate the validity of the model. The resulting data were plotted as response surface and contour plots to illustrate the relationship between the response and experimental levels of each variable used in this study.

## Results and Discussion

### Setting the Printing Condition Range of Variables

Prior to using an experimental statistical method for optimization, the printing condition range of variables was set. Print deviations of height, width, and length dimensions of the printed sample were calculated as the difference between the design dimensions and experimental dimensions measured using a vernier caliper to determine the printing accuracy affected by the variables : BW concentration and IL.

**Fig. 2:**
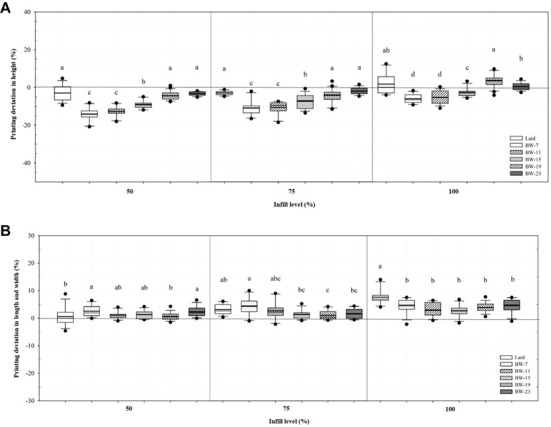
Dimensional deviation of 3D printed products. (A) the deviation of the height dimensions of printed samples, (B) the deviations in length and width of printed samples. A positive value of the deviation represents expansion of the object, and a negative value represents reduction of the object.

**Fig. 3:**
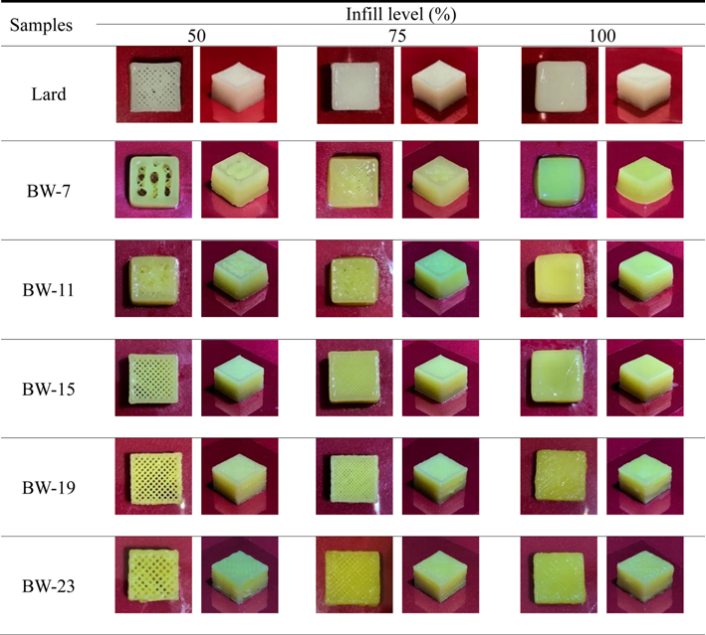
Appearance of 3D printed products.

Deviation of the height dimension of printed samples ranged from -14.12 to 3.53%, which seemed to marginally affect the visual appearance (Figs. 2A and 3). In addition, since most of the samples had negative values, a "thinner" tendency for decreases in height relative to the designed model was observed, which may be related to compressive deformation of the printed sample due to gravity [5]. BW density was observed to affect the height deviation, and generally, as the BW density increases, the deviation value decreases, indicating that printing accuracy increases. This observation is explained by the elastic rheological behavior of lard and high concentration BW oleogels. Deviation of the height dimension of lard was the smallest among the BW oleogel samples, showing the highest printing accuracy similar to that of BW-19. In the case of BW-7, -11, and -15 samples, it was observed that as the IL increases, the deviation value decreases and the printing accuracy increases, which may be due to the formation of a sturdy structure that supports the printed sample to prevent collapse as the IL increases. In contrast, the lard, BW-19 and -23 samples exhibited positive values at an IL of 100%, indicating an expanded shape of the object, which may be related to the ‘die swell behavior’ effect due to the robust viscoelastic properties of the above samples. This behavior reflects the fact that as viscoelastic fluid is extruded from the nozzle, the binding force on the tip wall is removed and can expand to a diameter larger than the nozzle diameter [38].

In the length and width dimensional characteristics of the printed samples (Fig. 2B), the deviations in length and width of these samples showed a tendency for ‘fatter,’ somewhat positive values, which were very similar to the shape of the designed model. The highest deviations in length and width were observed in BW-7, which can be accounted for by the high spreadability due to the viscous rheological properties of this sample. In addition, as the BW concentration increased, the overall diameter variation increased, which can be explained by the “die expansion shape” of the sample with high viscoelasticity, such as height variation. Therefore, BW-15 showed the lowest deviation value of the diameter and the highest printing accuracy. In general, no significant effect of IL on the deviation of length and width was noticed. However, it was observed that in the cases of lard, BW-19, and -23, as the IL decreased, the deviation value decreased, and the printing accuracy increased.

Overall, the lard, BW-15, -19, and -23 samples were similar to the designed virtual model, indicating that food 3D printing can achieve relative accuracy with personalized designs (Figs. 2, 3).

### Model Fitting of Texture Parameters

A CCFC experimental design was adopted to select the most optimal conditions for 3D structure printing with lard textural properties (HA, CO, and AD) using BW oleogel alternative fat. Before the CCFC, a pre-experiment was performed with regard to BW concentration and IL in order to set the variables (Fig. 2). Condition combinations and ranges of variables were developed to conduct the CCFC (Table 1).

Eqs. (3)–(5) represent the quadratic equations of the HA, CO, and AD models. The *R*^2^ (0.984 for HA, 0.948 for CO, 0.991 for AD), *R*^2^_adj_ (0.911 for HA, 0.911 for CO, 0.985 for AD) and *R*^2^_pred_ (0.850 for HA, 0.850 for CO, 0.955 for AD) values showed good agreement (Table 3) and were suitable to represent the actual relationship between the experimental factors and response.

$$\text{HA (g) = 202.0-34}{\text{.09X}}_{\text{1}}\text{-0}{\text{.38X}}_{\text{2}}\text{+0}{\text{.952X}}_{\text{1}}^{\text{2}}\text{-0}{\text{.0140X}}_{\text{2}}^{\text{2}}\text{+0}{\text{.2553X}}_{\text{1}}{\text{X}}_{\text{2}}$$ (3)

$$\text{CO=0.167-0}{\text{.0215X}}_{\text{1}}\text{+0}{\text{.01675X}}_{\text{2}}\text{-0}{\text{.00044X}}_{\text{1}}^{\text{2}}\text{-0}{\text{.000099X}}_{\text{2}}^{\text{2}}\text{+0}{\text{.000125X}}_{\text{1}}{\text{X}}_{\text{2}}$$ (4)

$$\text{AD (g.s) =1276+108}{\text{.6X}}_{\text{1}}\text{+19}{\text{.53X}}_{\text{2}}\text{-1}{\text{.537X}}_{\text{1}}^{\text{2}}\text{-0}{\text{.0355X}}_{\text{2}}^{\text{2}}\text{-1}{\text{.329X}}_{\text{1}}{\text{X}}_{\text{2}}$$ (5)

In the HA and AD models, the linear term of the BW concentration (X_1_) and IL (X_2_) together with the interactions BW × IL were shown to be highly significant factors (*p* < 0.001). On the one hand, the CO model was observed as a highly significant factor only in the linear terms of BW and IL (*p* < 0.001). The Eqs. (3–5) models effectively account for the changes in HA, CO, and AD as a function of BW concentration and IL.

**Fig. 4:**
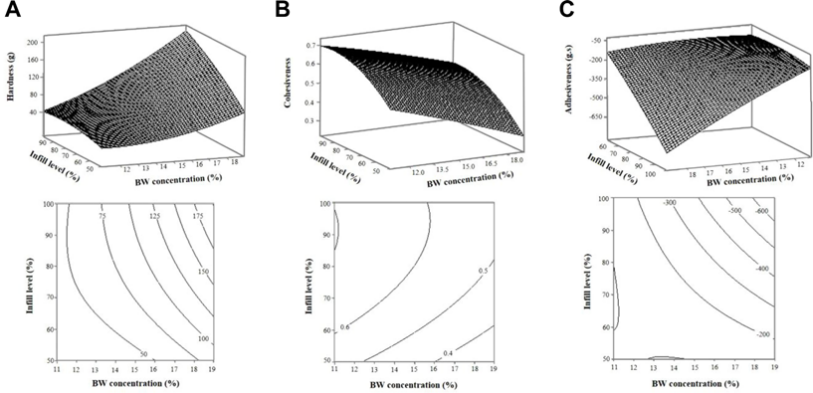
Response surface and contour plots describing the effects of BW concentration and IL on the HA, CO, and AD of 3D printed products. A, hardness of 3D printed products; B, cohesiveness of 3D printed products; C, adhesiveness of 3D printed products.

Fig. 4 visually shows the effect of BW concentrations and IL on HA, CO, and AD of the printed products. Within the studied range, HA was observed to increase rapidly with increasing BW concentration and slowly increase with increasing fill level (Fig. 4A). This result was expected because the elastic rheological properties increased with increasing BW concentration (data not shown). Additionally, an increase in IL was expected because it reflects a larger amount of oleogel being extruded to fill the internal structure of the sample. The influences of material concentrations and printing filling level on the hardness of the printed samples were similar to results reported in previous studies [5, 34]. In addition, the change in hardness increased as both the BW concentration and IL increased, indicating that the hardness was influenced by the interaction of these two variables, as shown in the Eq. (3) model.

In the case of cohesiveness, it was observed that cohesiveness decreased as the BW increased, contrary to HA (Fig. 4B). Cohesiveness tends to decrease with increasing hardness (proportional to A1) in the ratio of the second compression cycle (A3) to the first compression cycle (A1). It can be reasoned that the harder BW oleogels initially had a more resilient structure, but once compressed they became more irreversible deformations [5].

Adhesiveness is the negative force required for the sample to separate from the compression plunger, and high negative values indicate strong adhesion [37]. A linear increase in the negative values of adhesiveness was observed with increasing BW concentration and IL (Fig. 4C), which may result because it is associated with an increased complex viscosity (rheological parameter) with increasing BW concentration [39]. The increase in infill levels was expected as an increase in oleogel extrusion volume. In addition, the adhesiveness strength obtained its maximum value when both the BW concentration and IL were increased, and as shown in the model equation, it was found that adhesion was influenced by the interaction of these two variables.

### Optimization and Validation of BW-Based Oleogel Alternative Fat 3D Printing Structure

**Fig. 5:**
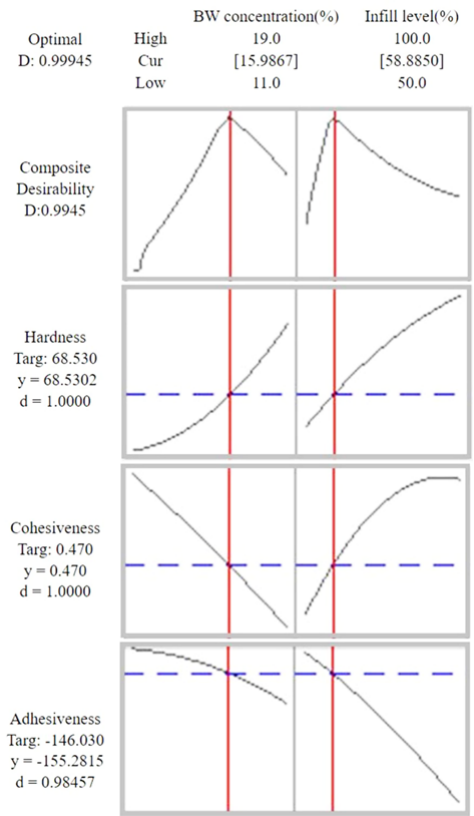
Multiple response optimization plots.

To produce optimal BW oleogels with textural properties (HA, CO, and AD) similar to lard, multiple reaction optimization was performed to determine a set of satisfactory preparation parameters that me*et al*l the demands imposed on the reaction parameters [5]. The target values for HA, CO, and AD were fixed at 68.53, 0.47, and -146.03, respectively, which are the lard values at the 75% infill level, such that the preparation parameters and responses can take arbitrary values within the analyzed range. As a result of multiple reaction examples, the best set values were found to be BW = 15.99%, IL = 58.89%, and the according D value was 0.9945 (Fig. 5). These optimal requirements were used in texture analysis to validate the predictive model.

As shown in Table 4, the properties of printed BW oleogels obtained with the optimized parameters gave almost identical values of HA and CO from lard with no statistically significant difference. In addition, the AD was slightly higher, but was within the range of the predictive model, and there was no statistically significant difference. The BW oleogel was able to imitate the textural properties of lard, and the predictive model satisfactorily described the actual behavior.

Based on the BW oleogel composition and the printing parameter IL, we performed 3D printing optimization of alternative fat with lard textural properties (HA, CO, and AD). We found that 3D printing was able to successfully provide the designed porous structure using the BW-15 sample. The dimensional properties of lard printed with ILs of 50 and 75% and the BW-15 sample were designed with similar accuracy. The textural properties were affected by BW concentration and IL, with the BW concentration imparting the highest contribution. As the BW increased, HA and AD were found to increase in an absolute curve as opposed to CO. As IL increased, HA, CO, and AD were found to increase in a curve and then decrease slightly. In addition, the interaction of BW and IL had a noteworthy influence on all responses except CO. The BW oleogel printed with an optimization preparation variable showed no significant difference in HA, CO, and AD compared to the properties of lard printed with 75% IL. Thus, we concluded that the goal of multiple reaction optimization was substantially achieved. Additionally, the multi-response mathematical model obtained through this study enables custom printing of BW oleogels. We emphasize that the BW concentration and printing parameters can modify the textural properties of printed products, and therefore this printed BW oleogel has the potential to replace lard, which is expected to provide similar texture and sensory properties of fat for materials in need of fat replacement. Future work will carry out complex marbling-related studies with the use of protein materials based on real meat.

## Figures and Tables

**Table 1:** Experimental matrix for central composite face-centered (CCFC) experiment and response observations under different experimental conditions.

Run	Codes^a^		Experimental factors^b^		Response variables^c^		
		
	X_1_	X_2_	BW(%)	IL(%)	HA(g)	CO	AD(g.s)
1	-1	+1	11	100	45.68	0.69	-132.47
2	-1	-1	11	50	29.52	0.53	-101.96
3	0	0	15	75	84.98	0.55	-214.25
4	0	0	15	75	91.66	0.59	-238.07
5	0	-1	15	50	44.84	0.42	-101.52
6	+1	0	19	75	160.83	0.45	-370.88
7	0	0	15	75	81.12	0.60	-274.25
8	0	0	15	75	76.47	0.64	-218.84
9	0	+1	15	100	105.78	0.61	-366.49
10	0	0	15	75	77.83	0.55	-203.04
11	+1	+1	19	100	198.68	0.53	-709.01
12	+1	-1	19	50	90.42	0.32	-147.09
13	-1	0	11	75	38.12	0.69	-101.96

All response variables were repeated at least 3 times.

^a^X_1_, BW concentration; X_2_, infill level

^b^BW, concentration of beeswax; IL, infill level

^c^HA, hardness; CO, cohesiveness; AD, adhesiveness

**Table 2:** Operating parameters of the 3D printer.

Factors		Unit	Value
Printing speed		mm/min	700
Nozzle diameter		μm	400
Extrusion multiplier		-	0.02
Filament diameter		μm	300
Flow late		%	100

Layer height	Primary	μm	400
	First	μm	320

Printing time	50% infill level	min	11
	75% infill level	min	15
	100% infill level	min	18

**Table 3:** Analysis of variance (ANOVA) for fitted quadratic polynomial models for hardness (HA), cohesiveness (CO), and adhesiveness (AD).

Source	Sum of squares	Degree of freedom	Mean squares	*F*-value	*p*-value
*Hardness (HA)*					
Model	27418.4	5	5483.7	121.17	0.000
Linear	24139.9	2	12070.0	266.69	0.000
X_1_	17779.0	1	17779.0	392.84	0.000
X*2*	6360.9	1	6360.9	140.55	0.000
Square	627.3	2	336.2	7.43	0.019
X_1_X_1_	640.6	1	640.6	14.15	0.007
X_2_X_2_	220.5	1	220.5	4.87	0.063
2-Way Interaction	2606.1	1	2606.1	57.58	0.000
X_1_X_2_	2606.1	1	2606.1	57.58	0.000
Error	316.8	7	45.3		
Lack-of-Fit	166.7	3	55.6	1.48	0.347
Pure Error	150.1	4	37.5		
Total	27735.2	12			
R^2^	0.9486	R^2^_Adj_	0.9118	R^2^_Pred_	0.8502
*Cohesiveness (CO)*					
Model	0.1286	5	0.0257	25.82	0.000
Linear	0.1133	2	0.0571	57.37	0.000
X_1_	0.0620	1	0.0620	62.26	0.000
X*2*	0.0523	1	0.0523	52.47	0.000
Square	0.0137	2	0.0068	6.87	0.022
X_1_X_1_	0.0001	1	0.0001	0.14	0.721
X_2_X_2_	0.0106	1	0.0106	10.68	0.014
2-Way Interaction	0.0006	1	0.0006	0.63	0.454
X_1_X_2_	0.0006	1	0.0006	0.63	0.454
Error	0.0070	7	0.0010		
Lack-of-Fit	0.0013	3	0.0004	0.29	0.830
Pure Error	0.0057	4	0.00014		
Total	0.1356	12			
R^2^	0.9486	R^2^_Adj_	0.9118	R^2^_Pred_	0.8502
*Adhesiveness (AD)*					
Model	330190	5	66038	160.71	0.000
Linear	254703	2	127351	309.92	0.000
X_1_	132186	1	132186	321.69	0.000
X*2*	122517	1	122517	298.16	0.000
Square	4883	2	2442	5.94	0.031
X_1_X_1_	1670	1	1670	4.06	0.084
X_2_X_2_	1358	1	1358	3.30	0.112
2-Way Interaction	70604	1	70604	171.82	0.000
X_1_X_2_	70604	1	70604	171.82	0.000
Error	2876	7	411		
Lack-of-Fit	1419	3	473	1.30	0.390
Pure Error	1457	4	364		
Total	333067	12			
R^2^	0.9914	R^2^_Adj_	0.9852	R^2^_Pred_	0.9554

**Table 4:** Multi-response optimization of 3D printed BW oleogels.

	Experimental factors^a^		Response variable^b^		

	BW (%)	IL (%)	HA (g)	CO	AD (g.s)
Lard	0.00	75.00	68.53 ± 10.20^a^	0.47 ± 0.33^a^	-146.03 ± 21.39^a^
Predictive model	15.99	58.89	68.53	0.47	-155.28
Printed BW oleogels	15.98	59.00	68.41 ± 6.99^a^	0.48 ± 0.05^a^	-155.44 ± 11.71^a^

All tests were repeated 3 times. Values are presented as mean ± SD.

^a^BW, concentration of beeswax; IL, infill level

^b^HA, hardness; AD, adhesiveness; CO, cohesiveness; Different letters within the same column indicate significant differences at *p* < 0.05 (ANOVA by Duncan test).
